# A rare case report of immunoglobulin G4-related sclerosing mesenteritis and review of the literature

**DOI:** 10.1097/MD.0000000000022579

**Published:** 2020-10-09

**Authors:** Zhicheng Liu, Yan Jiao, Liang He, Helei Wang, Daguang Wang

**Affiliations:** a Department of Gastroenterological Surgery; b Department of hepatobiliary and pancreatic surgery, The First Hospital of Jilin University, Changchun, Jilin, China.

**Keywords:** autoimmune disease, immunoglobulin G4-related disease, immunoglobulin G4-related sclerosing mesenteritis, sclerosing mesenteritis

## Abstract

**Introduction:**

Immunoglobulin G4 (IgG4)-related disease (IgG4-RD) is a rare autoimmune disorder involving 1 or multiple organs, most commonly the pancreas, lacrimal glands, and salivary glands. However, IgG4-related sclerosing mesenteritis (SM) involving the small-bowel mesentery is rare. Given that IgG4-related SM usually mimics the imaging characteristics of mesenteric malignancies, its preoperative diagnosis remains challenging. In addition, no specific consensus has been reached regarding the treatment of IgG4-related SM. Therefore, a better understanding of the characteristics, treatment, and prognosis of IgG-related SM is urgently needed. Herein, we report a rare case of IgG-related SM.

**Patient concerns:**

A 67-year-old man was admitted to our hospital after incidental detection of an abdominal mass on ultrasound imaging, although he reported being generally well. The findings on triple-phase abdominal computed tomography were highly consistent with a malignant mesenteric tumor.

**Diagnoses:**

The hallmark histopathological features along with elevated levels of IgG4 (145 mg/dL) and imaging findings were indicative of IgG-related SM.

**Interventions:**

The patient was treated surgically. Postoperative histopathological examinations exhibited tissue infiltration with lymphocytes and IgG4-positive plasma cells, as well as fibrosis.

**Outcomes:**

Ten days after surgery, the patient was discharged from the hospital, and did not show any clinical sign of IgG-related SM within 1-year follow-up.

**Conclusion:**

This case highlights the mesentery as an uncommon site of involvement as well as how early IgG-related SM can be completely asymptomatic. Thus, this study has advanced our knowledge of IgG-related SM and may improve treatments for similar conditions.

## 1 Introduction

IgG4-related disease (IgG4-RD) is a rare autoimmune disease characterized chiefly by tissue infiltration by lymphocytes, IgG4-positive plasma cells, fibrosis as a response to persistent chronic inflammation, and a tendency toward mass formation.^[1–4]^ IgG-RD can involve 1 or multiple organs in the human body. The pancreas, lacrimal glands, and salivary glands are the most common sites of involvement, whereas the mesentery is rarely affected.^[2–6]^ To date, only 13 cases of IgG4-related sclerosing mesenteritis (SM) have been confirmed by characteristic histological findings to be manifestations of IgG4-RD.^[7–18]^ Because it has imaging features similar to malignant tumors and there is a lack of specific markers, it is extremely challenging for physicians to make an accurate diagnosis of IgG4-related SM without histological examination of biopsy or resected specimens. Thus, misdiagnosis is unfortunately quite common. Notably, the current treatment options are based on the recommendations for the management of IgG4-RD as developed by a panel of experts, and no guideline specific to IgG4-related SM has been realized. In addition, the long-term clinical outcomes of IgG-related SM remain unclear. As such, a better understanding of the characteristics, treatment, and prognosis of IgG-related SM is required.

In this case study, we present an uncommon case of IgG-related SM in a 67-year-old man. We also performed a literature review and summarized the reported cases of IgG-related SM.

## 2 Case presentation

A 67-year-old male patient was admitted to our hospital 10 days after ultrasound imaging revealed a mass inside the abdominal cavity during a routine physical examination. He had no medical history of abdominal surgery.

**Figure 1:**
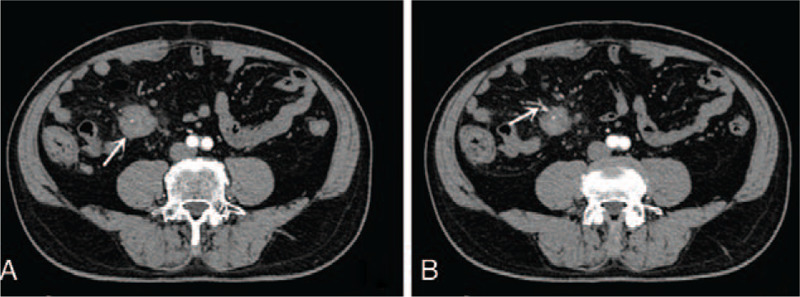
Pre-operational computed tomography (CT) findings. (A) CT revealed slight enhancement of a mass (approximately 4 cm in diameter) at the root of the small-bowel mesentery (SBM) (arrow), and multiple nodular satellite foci were observed around the mass. (B) The branches of the mesenteric artery passed through the mass. SBM = small-bowel mesentery.

**Figure 2:**
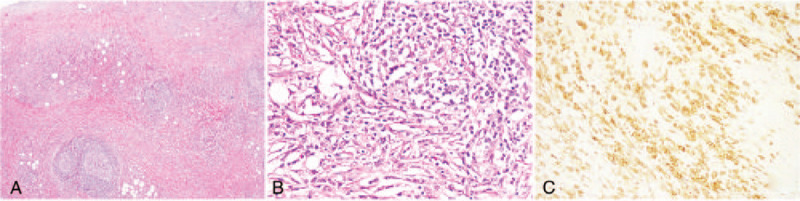
Post-operational histological examinations. (A) Increases in the lymphoid follicles and collagen fibers (hematoxylin and eosin [H&E], 10x). (B) Tissue infiltration by lymphocytes (H&E, 200x). **(C)** Tissue filtration with IgG4-positive plasma cells (H&E, 200x).

Laboratory examinations showed that his white blood cell and eosinophil counts were in the normal ranges. Triple-phase abdominal computed tomography (CT) identified an uneven, slightly enhanced mass approximately 4 cm in diameter at the root of the small-bowel mesentery in the lower right abdomen, which was surrounded by multiple nodular satellite foci (Fig. 1A). In addition, CT imaging showed mesenteric vessels running through the mass (Fig. 1B). No obvious abnormality in the liver, biliary tract, or pancreas was detected (data not shown). Because these CT findings highly mimicked a malignant mesenteric tumor and mesenteric biopsy seemed high risk and inappropriate, the patient underwent surgical treatment. During the procedure, a number of hard masses were observed with no clearly defined boundary located in the mesentery nearly 100 cm to the ileocecal junction, and the largest 1 was approximately 4 cm in diameter. Subsequently, the mesenteric masses along with a fragment of the small intestine were resected. Postoperative histological examinations indicated sclerosing lesions, fibrosis (Fig. 2A), as well as tissue infiltration by lymphocytes and plasma cells (Fig. 2B). Further, immunohistochemistry (IHC) examination showed positive staining for IgG4 in the plasma cells (>50 × high-power field with an IgG4+/IgG+ ratio of plasmacytic infiltration >40% (Fig. 2C). IHC analysis also detected positive staining for CD138 (+). The serum level of IgG4 was 145 mg/dL. Based upon the abdominal CT features, intraoperative findings, postoperative histopathological and laboratory examinations, the diagnosis of IgG4-related SM was made and confirmed in our patient.

The patient was discharged from hospital 10 days after surgery. At the 1-year follow-up visit, the patient did not show any clinical sign of recurrent IgG-related SM. However, it must be noted that postoperative ultrasound imaging revealed 2 masses on the left side of the parotid glands, suggesting involvement of the parotid glands. Methylprednisolone tablets (24 mg/d) were prescribed, but the patient declined the hormone therapy. There was no apparent change in the parotid masses at the 1-year follow-up.

## 3 Discussion

IgG4-RD is a recently recognized autoimmune disease, and the precise etiology and pathological mechanisms remain unknown.^[1]^ The pancreas, lacrimal glands, and salivary glands have been described as the most commonly affected organs. In this case study, we described an uncommon case of IgG4-related SM with the small-bowel mesentery as the involved site.

The diagnosis of IgG4-SD is not straight-forward; indeed, it is extremely challenging to make an accurate diagnosis without pathologically assessment of biopsy or resected specimens from an involved organ. Although there is no internationally accepted consensus on diagnostic criteria for IgG4-SD, the following Japanese criteria that were established in 2011 by a Japanese consensus group are widely used:

(1) Clinical examination showing characteristic diffuse/localized swelling or masses in single or multiple organs;
(2) Hematological examination shows elevated serum IgG4 concentrations (135 mg/dl);
(3) Histopathologic examination shows

a) Marked lymphocyte and plasmacyte infiltration and fibrosis,
b) Infiltration of IgG4+ plasma cells: ratio of IgG4+/IgG+ cells > 40% and >10 IgG4+ plasma cells/ high-power field.^[2]^

Notably, the serum IgG4 level is elevated in a majority of IgG-RD patients, but not in all such patients.^[3]^ In fact, no specific serum marker has been determined to be useful for the diagnosis of IgG4-RD.^[3–6]^ In clinical practice, IgG4-RD is usually diagnosed after tissue biopsy or resected specimens have been pathologically evaluated. In accordance with the Japanese criteria, the diagnosis of IgG4-Rd is confirmed if the 3 criteria are satisfied. In the present case, the histopathology of resected tissue specimens showed the hallmark features of IgG4-RD, together with an elevated level of IgG4 (145 mg/dL), and imaging findings of mass lesions, which together suggested IgG-related SM. Moreover, we also ruled out malignant tumors in the postoperative pathological examination of resected tissue specimens. The diagnosis of IgG-related SM was finally made and confirmed in our patient.

To better understand the characteristics, course, treatment, and prognosis of IgG-related SM, we conducted a literature search of PubMed using the keywords “IgG4” and “mesenteritis” and retrieved a limited number of reports on IgG4-related SM cases. Of these studies, 12 cases fulfilled the Japanese criteria for the diagnosis of IgG4-related SM. The major of findings from these cases as well as ours are summarized in Table 1.^[7–18]^ The reported IgG4-related SM patients included 8 males and 5 females with an age range of 7 to 82 years (mean age, 56 years). Chronic abdominal pain was commonly described in those cases, while other clinical manifestations include acute local peritonitis and intestinal obstruction caused by invasion of the intestinal wall. The IgG4-related SM in these cases involved a variety of sites, including the mesentery of the terminal ileum, the mesentery at the root of the small intestine, and the entire small-bowel mesentery. Seven patients had a medical history of abdominal surgery, and 1 patient had a history of tuberculosis, implicating a speculative association between previous abdominal surgery and abdominal tuberculosis and IgG4-related SM.^[6]^ It was noticed that 6 patients had serum levels of IgG4 ≥135 mg/dL, and this is consistent with the fact that IgG4 is not always elevated in IgG4-RD. Thus, the cutoff value of ≥135 mg/dL is not reliable for the diagnosis of IgG4-RD. IgG4-RD is a highly heterogeneous disease, and this characteristic has posed considerable challenges in the diagnosis and differential diagnosis of the disease. CT imaging is commonly used for the detection and localization of abdominal masses. All the IgG4-related SM patients had undergone abdominal CT, and analysis of the CT findings revealed the following specific CT features of IgG4-related SM:

(1) round or oval mesenteric solid tumors with ill-defined margins located mainly at the root of the small-bowel mesentery or the distal ileum mesentery;
(2) slightly enhanced on CT;
(3) likely surrounded by enlarged lymph nodes;
(4) invasion of adjacent intestinal wall or other organs; and
(5) mesenteric vessels running through the mass.

As IgG4-related SM usually mimics the imaging characteristics of malignancies, misdiagnosis as mesenteric cancer is very common. In a previous study, the fat-ring sign (fat halo sign) and pseudocapsule sign were described as specific CT characteristics of IgG4-related SM.^[19]^ However, we did not observe these 2 changes on CT in the present patient. Among the 13 IgG4-related SM patients listed in Table 1, 5 cases underwent ^18^F-FDG PET-CT and 4 patients showed an increase in radioactive tracer uptake at the lesion sites. However, ^18^F-FDG PET-CT in combination is also limited for the diagnosis and differential diagnosis of IgG4-RD.^[20]^ The diagnosis of IgG-SM must rely on the histopathological findings of biopsy or resected specimens of the involved organs, during which precaution must be taken to avoid misdiagnosis by excluding malignancies. Typically, a resected biopsy is required for the diagnosis, as needle biopsy may cause intra-abdominal bleeding. For example, only 2 IgG4-related SM cases underwent a needle biopsy for pathological diagnosis, and a majority of the patients were diagnosed with IgG4-related SM based on analysis of resected specimens of involved organs (Table 1). A summary of the preoperative radiological images of the IgG4-related SM cases in Table 1 may promote a better understanding of the features of this disease on abdominal imaging. Unfortunately, the serum markers and imaging methods developed to date are unreliable. Despite the relatively lesser importance of imaging for the diagnosis of IgG-RD, such information might help when a tissue biopsy should be performed. For instance, in our case abdominal CT imaging showed blood vessels passing through the mass lesions and specific location, which indicates an increased risk of developing lower gastrointestinal bleeding and perforation during the biopsy procedure.

**Table 1:**
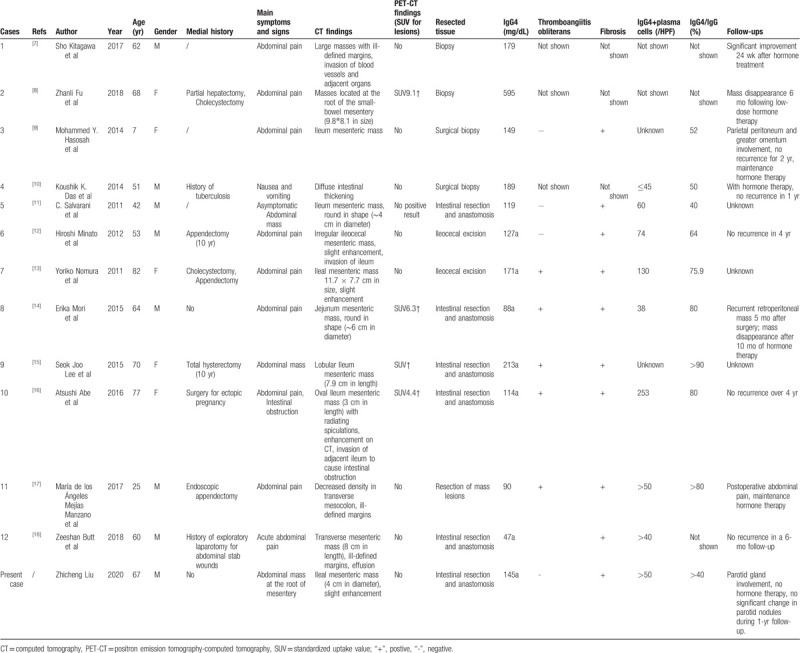
Summary of the characteristics of all cases of IgG4-related sclerosing mesenteritis found in the literature.

At present, glucocorticoid therapy is recommended as the first-line treatment for IgG4-RD. The goal of treatment is to prevent tissue fibrosis and the related dysfunction of affected organs. The international guideline on the management of IgG4-RD has been established.^[19]^ However, there is no definitive treatment for IgG4-RD, in particular, IgG4-related SM. Glucocorticoid therapy is associated with the following limitations: high overall relapse rate as high as 30%,^[20]^ relatively long course of treatment, and adverse effects. Also notable is that IgG4-RD is associated with a high risk of progression into malignant tumors. Greenbaum and colleagues recently suggested that, for autoimmune disease-related mass lesions, especially those suspected of malignant transformation, surgical excision should be considered.^[21]^ Given that it is a considerable challenge to distinguish between IgG4-related SM and malignant tumors. For the above reasons, we are more inclined to manage such cases with a surgical procedure. In this case, we adopted a surgical approach for the treatment of IgG4-related SM and prescribed glucocorticoids as the treatment plan for an involvement of parotid glands. The patient did not have any clinical sign of recurrent IgG-related SM at 1 year after surgery. However, it remains uncertain whether IgG4-related SM patients who have not undergone complete resection of SM will need glucocorticoids to prevent recurrence of IgG4-related SM, as conflicting results have been reported.^[19,20]^ Additional investigations of the long-term clinical outcomes will be needed to understand optimal post-operative treatment for IgG-SM. Moreover, there are currently no individual risk factors associated with recurrent IgG4-related SM that could be used for prediction of recurrence.

In summary, IgG4-related SM is rare, and its preoperative diagnosis is extremely challenging. This case study demonstrates that although an uncommon site, the mesentery can be a site of involvement, and in the early stages, IgG-related SM can be completely asymptomatic. This case study and literature review have advanced our knowledge of IgG-related SM and can be applied in its diagnosis, treatment, and prognosis.

## Author contributions

**Conceptualization:** Daguang Wang.

**Funding acquisition:** Daguang Wang.

**Investigation:** Liang He.

**Resources:** Zhicheng Liu.

**Supervision:** Helei Wang.

**Writing – original draft:** Zhicheng Liu.

**Writing – review & editing:** Yan Jiao.

## Abbreviations

Abbreviations: CT = computed tomography, IgG4-RD = IgG4-related disease, SM = sclerosing mesenteritis.
